# *Sirt1* is regulated by miR-135a and involved in DNA damage repair during mouse cellular reprogramming

**DOI:** 10.18632/aging.103090

**Published:** 2020-04-26

**Authors:** Andy Chun Hang Chen, Qian Peng, Sze Wan Fong, William Shu Biu Yeung, Yin Lau Lee

**Affiliations:** 1 Department of Obstetrics and Gynaecology, The University of Hong Kong, Hong Kong SAR, China; 2 Shenzhen Key Laboratory of Fertility Regulation, The University of Hong Kong Shenzhen Hospital, Shenzhen, China

**Keywords:** mouse induced pluripotent stem cells, cellular reprogramming, Sirt1, miR-135a, DNA damage repair

## Abstract

*Sirt1* facilitates the reprogramming of mouse somatic cells into induced pluripotent stem cells (iPSCs). It is regulated by micro-RNA and reported to be a target of miR-135a. However, their relationship and roles on cellular reprogramming remain unknown. In this study, we found negative correlations between miR-135a and *Sirt1* during mouse embryonic stem cells differentiation and mouse embryonic fibroblasts reprogramming. We further found that the reprogramming efficiency was reduced by the overexpression of miR-135a precursor but induced by the miR-135a inhibitor. Co-immunoprecipitation followed by mass spectrometry identified 21 SIRT1 interacting proteins including KU70 and WRN, which were highly enriched for DNA damage repair. In accordance, *Sirt1* activator resveratrol reduced DNA damage during the reprogramming process. *Wrn* was regulated by miR-135a and resveratrol partly rescued the impaired reprogramming efficiency induced by *Wrn* knockdown. This study showed *Sirt1*, being partly regulated by miR-135a, bound proteins involved in DNA damage repair and enhanced the iPSCs production.

## INTRODUCTION

The reprogramming of somatic cells into induced pluripotent stem cells (iPSCs) is one of the most innovative scientific breakthroughs in the field of regenerative medicine. In 2006, Takahashi and Yamanaka introduced *c-Myc*, *Klf4*, *Oct4* and *Sox2* (MKOS) into mouse adult fibroblasts and successfully converted them into iPSCs [1]. Similar to embryonic stem cells (ESCs), iPSCs are pluripotent and give rise to different cell lineages upon teratoma formation, in chimeric and tetraploid embryos production [2]. Since then, iPSCs have become an important tool for patient-specific cell therapy and disease modeling.

Chromatin remodeling occurs in the initiation phase of reprogramming, implying that chromatin modifying enzymes are involved in regulating the process [3]. Genes or small molecules related to chromatin remodeling enhance reprogramming efficiency. For instance, DNA methyltransferase inhibitor, histone methyltransferase G9a inhibitor [4–6], and histone deacetylase inhibitor valproic acid (VPA) [7] can greatly improve the efficiency of iPSCs production. We have previously reported the involvement of another histone deacetylase, *Sirt1*, in reprogramming. Activation of *Sirt1* by resveratrol (RSV) facilitates the reprogramming efficiency of mouse fibroblasts [8].

MicroRNAs (miRNAs) are small non-coding RNAs important for maintaining pluripotency in ESCs [9, 10]. In the context of reprogramming, miR-302 enhances the reprogramming efficiency [11]. *Sirt1* can be regulated by miR-34a. We [8] and others [12] demonstrated that force expression of miR-34a reduced while inhibiting miR-34a enhanced reprogramming efficiency. Blockade of miR-195 that also targets *Sirt1* increases reprogramming efficiency in old skeletal myoblasts [13]. Successful iPSC formation can be obtained by direct transfection of mature miRNAs (miR-200c, miR-302s and miR-369s) [14]. Although iPSCs can be obtained using different approaches, the epigenetic and molecular events underlying cell fate conversion are not fully understood.

Here we demonstrated that miR-135a inhibited reprogramming efficiency through targeting *Sirt1*. We also identified SIRT1 interacting proteins WRN and KU70 that actively participated in the initiation phase of reprogramming through repairing DNA damage during reprogramming. Our data provide an understanding on the role of miR-135a-*Sirt1* axis and the *Sirt1* interacting partners during reprogramming.

## RESULTS

### miR-135a impeded reprogramming efficiency partly through inhibiting *Sirt1*

Reprogramming system was established by transducing 1° mouse embryonic fibroblast (MEFs) with the doxycycline-inducible polycistronic lentiviral vector (4F2A) for transcription of *Oct4*, *Sox2*, *Klf4* and *c-Myc* (Addgene #20231 & #20342). After 5 days of DOX treatment, immunocytochemistry staining showed slight increase in proportion of OCT4-positive cells with increased multiplicity of infection (MOI) [Supplementary Figure 1]. To avoid large numbers of transgenes inserted into the host genome, a MOI of 10 was used for subsequent assays. In addition to 1° MEFs, 2° MEFs containing the DOX-inducible reprogramming factors [2] were also used in this study. The reprogrammed colonies from both 1° and 2° MEFs showed positive alkaline phosphatase staining. Furthermore, the iPSC colonies formed from 1° MEFs were stained positively for pluripotent markers SSEA-1 and NANOG [Supplementary Figure 1], which agreed with our published data showing positive SSEA-1 and NANOG staining in iPSC formed from 2° MEFs [8].

**Figure 1:**
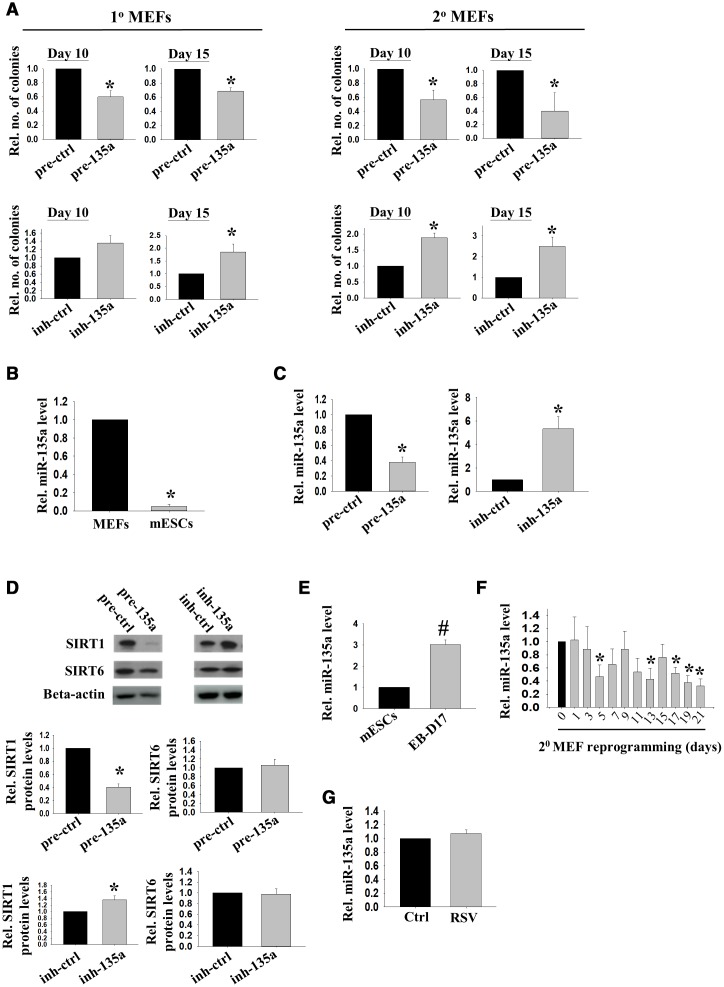
**miR-135a impeded reprogramming efficiency partly through inhibiting *Sirt1*.** (**A**) The effects of miR-135a precursor or inhibitor on relative number of iPSC colonies formed from 1° (n=4) or 2° (n=7) MEFs on D10/15 after Doxycycline (DOX) induction. miRNA precursor (pre-ctrl) and inhibitor (inh-ctrl) with scrambled sequence were used as controls (*:p<0.05; t-test). (**B**) The relative expression levels of miR-135a in MEFs and mESCs (n=3; *: p<0.05; t-test). (**C**) The relative expression levels of miR-135a in 1° MEFs after transfecting with miR-135a precursor (pre-135a) or inhibitor (inh-135a) for 72h (n=4; *:p<0.05; t-test). (**D**) The relative SIRT1 and SIRT6 protein levels in 1° MEFs after transfecting with miR-135a precursor (pre-135a) or inhibitor (inh-135a) for 72h (n=4; *:p<0.05; t-test). (**E**) The relative expression levels of miR-135a in mESCs and EBs at day 17 (EB-D17, n=3; #:p<0.001; t-test). (**F**) The relative miR-135a expression levels during reprogramming of 2° MEFs from day 0 to day 21 (n=3; *:p<0.05; t-test). (**G**) The relative expression levels of miR-135a in 1° MEFs after RSV treatment for 72h (n=4; t-test).

Reprogramming to pluripotency involves genome-wide chromatin remodeling [3]. *Sirt1,* a histone deacetylase regulated by miR-34a, facilitates reprogramming to mouse iPSCs [8]. *Sirt1* can also be regulated by miR-135a [15]. To study the roles of miR-135a in reprogramming, 1° and 2° MEFs were treated with precursor of miR-135a. The reprogramming efficiency was assessed by counting the number of colonies on day 10 and day 15 after DOX treatment. The results demonstrated that the precursor of miR-135a significantly down-regulated the reprogramming efficiency in 1° and 2° MEFs on both day 10 and day 15 (Figure 1A). To confirm the action of miR-135a, we determined the effect of its inhibitor on MEFs reprogramming and found that the inhibitor of miR-135a significantly enhanced the reprogramming efficiency on day 15 in both 1° and 2° MEFs (Figure 1A). Besides, the expressions of miR-135a in mESCs were significantly lower than that in the MEFs (Figure 1B), consistent with the possibility that miR-135a was a negative regulator of reprogramming. The relationship between miR-135a and *Sirt1* in reprogramming was studied. Quantitative PCR analysis demonstrated the levels of miR-135a were significantly down-regulated and up-regulated by the transfections of miR-135a inhibitor and precursor, respectively (Figure 1C). The precursor of miR-135a significantly reduced while the inhibitor significantly induced the SIRT1 protein levels in MEFs (Figure 1D). To demonstrate the specificity of miR-135a on *Sirt1*, we measured the levels of *Sirt6,* another SIRT family member which has common functions as *Sirt1* in stress resistance, vascular aging and cardiovascular disease. We found that miR-135a has no effect on SIRT6 protein levels [Figure 1D], suggesting the specificity of miR-135a on *Sirt1*. We previously showed that *Sirt1* was down-regulated upon mESCs differentiation and up-regulated upon reprogramming to pluripotency [8]. Here, we showed that miR-135a levels were significantly higher in spontaneously differentiating embryoid bodies (EBs) when compared to undifferentiated mESCs (Figure 1E); while its levels were down-regulated during MEFs reprogramming (Figure 1F). The effect of *Sirt1* activator (RSV) on miR-135a expression was followed, but it had no effect on miR-135a expression in the treated cells (Figure 1G).

### SIRT1 interacted with DNA repair proteins in the initiation phase of reprogramming

Our previous data demonstrated *Sirt1* deacetylated *p53*, leading to down-regulation of *p21* expression during reprogramming of MEFs [8]. *p53*/*p21* negatively regulates cell proliferation upon DNA damage [16, 17]. Consistently, our results here showed that inhibitor of miR-135a significantly enhanced the proliferation of MEFs [Supplementary Figure 2]. To further identify the SIRT1 interacting proteins during reprogramming, co-immunoprecipitation followed by mass spectrometry was followed.

**Figure 2:**
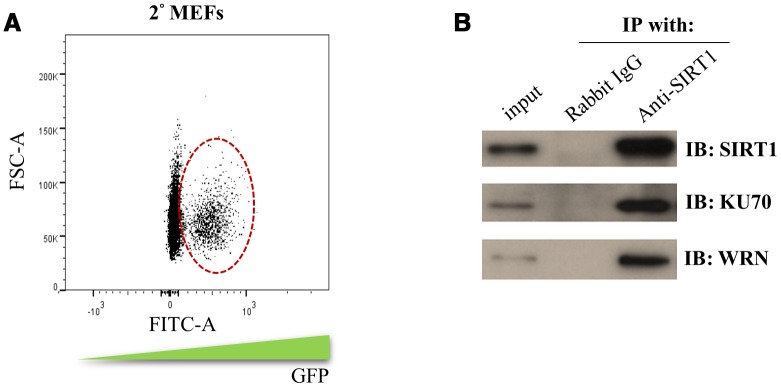
**Identification of the SIRT1 interacting proteins during initiation phase of reprogramming.** (**A**) 2° MEFs containing DOX inducible MKOS was sorted out by FACS according to the FITC gating indicated by circle dash line. (**B**) co-immunoprecipitation assay using SIRT1 antibody or rabbit IgG followed by Western blotting analysis (IB) using antibodies against SIRT1, KU70 and WRN.

Our 2° MEFs reprogramming system was derived from mouse embryonic fibroblasts (2uF/ 1B MEF) containing the doxycycline (DOX) inducible MKOS reprogramming factors. To have an efficient reprogramming system for isolating sufficient amount of SIRT1 interacting proteins, the cell population containing DOX-inducible vector was enriched by cell sorting of 2° MEFs (Figure 2A). The sorted 2° MEFs were subjected to DOX induction and co-immunoprecipitation to enrich the SIRT1 interacting proteins during the initiation phase (day 5) of reprogramming. The protein bands that were differentially enriched in the SIRT1 antibody group were cut and subjected to mass spectrometry protein identification. In total, 21 proteins with the total ion score C.I.% (http://www.matrixscience.com/) above 90 were identified (Table 1).

**Table 1:** Sirt1 interacting proteins during the initiation phase of miPSCs production identified by LC-ESI-Ion Trap mass spectrometry.

**Protein name**	**NCBI accession number**	**Protein MW**	**Score**
myosin-XVIIIa	gi|22094119	231791	1670
Kif21a	gi|6561827	176938	327
WRN protein	gi|7595900	159196	197
enhancer of mRNA-decapping protein 4	gi|31712002	151928	117
glutamyl-prolyl-tRNA synthetase	gi|148681120	166980	115
FH1/FH2 domain-containing protein 1	gi|269973931	130774	1784
DNA repair protein RAD50	gi|60392985	154533	1056
valyl-tRNA synthetase	gi|4590328	141523	1019
gamma-tubulin complex component 3	gi|39930567	104145	1057
gamma-tubulin complex component 2	gi|21362572	103796	608
heterogenous nuclear ribonucleoprotein U	gi|3329496	88635	151
gamma-tubulin complex component 5	gi|46560557	118815	135
dynamin-like 120 kDa protein, mitochondrial isoform 2 precursor	gi|19526960	111783	1009
double-strand break repair protein MRE11A	gi|9055282	80572	506
nedd-1 protein	gi|286103	71891	857
Stress-70 protein, mitochondrial	gi|14917005	73768	814
heat shock protein 70 cognate	gi|309319	71021	505
gamma-tubulin complex component 4	gi|23943924	76592	272
ATP-dependent RNA helicase DDX3X	gi|6753620	73455	117
X-ray repair cross-complementing protein 6	gi|145587104	69727	93
Elongation factor 1-delta	gi|13124192	31388	2929

The identified SIRT1 interacting proteins were subjected to gene ontology analysis using DAVID Bioinformatics Resources. Interestingly, the SIRT1 interacting proteins were enriched for gene ontology terms like DNA duplex unwinding, double-strand break repair and DNA repair (Table 2). In addition, pathway clustering analysis using Kyoto Encyclopedia of Genes and Genomes (KEGG) database indicated that two major DNA repair pathways, non-homologous end joining and homologous recombination, were enriched for SIRT1 interacting proteins (Table 2). Among them, Werner Syndrome RecQ Like Helicase (WRN) and X-ray repair cross-complementing protein 6 (XRCC6/KU70) were selected for further analysis. We confirmed by co-immunoprecipitation that WRN and KU70 bound with SIRT1 during the initiation phase (day 5) of 2° MEFs reprogramming (Figure 2B). The above results showed SIRT1 may form a protein complex with DNA repair proteins during the initiation phase of reprogramming.

**Table 2:** Gene ontology and KEGG pathway analysis of the enriched Sirt1 interacting proteins during the initiation phase of miPSCs production.

**GO term**	**Genes**	**Fold enrichment**	**p-value**
GO:0051415~interphase microtubule nucleation by interphase microtubule organizing center	Tubgcp4, Tubgcp5, Tubgcp3, Tubgcp2	574.0317	2.31E-08
GO:0007020~microtubule nucleation	Tubgcp4, Tubgcp5, Tubgcp3, Tubgcp2	215.2619	6.42E-07
GO:0032508~DNA duplex unwinding	Xrcc6, Rad50, Wrn, Mre11a	191.3439	9.34E-07
GO:0051298~centrosome duplication	Tubgcp4, Tubgcp5, Tubgcp3, Tubgcp2	156.5541	1.76E-06
GO:0007126~meiotic nuclear division	Tubgcp4, Tubgcp5, Tubgcp3, Tubgcp2	143.5079	2.31E-06
GO:0090307~mitotic spindle assembly	Tubgcp4, Tubgcp5, Tubgcp3, Tubgcp2	98.40544	7.40E-06
GO:0031122~cytoplasmic microtubule organization	Tubgcp4, Tubgcp5, Tubgcp3, Tubgcp2	95.67196	8.07E-06
GO:0006302~double-strand break repair	Xrcc6, Rad50, Wrn, Mre11a	54.66969	4.40E-05
GO:0000226~microtubule cytoskeleton organization	Tubgcp4, Tubgcp5, Tubgcp3, Tubgcp2	35.87698	1.55E-04
GO:0000723~telomere maintenance	Xrcc6, Rad50, Wrn	67.97744	7.98E-04
GO:0006974~cellular response to DNA damage stimulus	Xrcc6, Sirt1, Rad50, Wrn, Mre11a	10.25057	0.001035
GO:0006310~DNA recombination	Xrcc6, Rad50, Wrn	30.38992	0.003927
GO:0031860~telomeric 3' overhang formation	Rad50, Mre11a	430.5238	0.004417
GO:0006281~DNA repair	Xrcc6, Rad50, Wrn, Mre11a	10.83079	0.00492
GO:0000731~DNA synthesis involved in DNA repair	Sirt1, Wrn	215.2619	0.008816
GO:0032206~positive regulation of telomere maintenance	Rad50, Mre11a	172.2095	0.011009
GO:0000722~telomere maintenance via recombination	Rad50, Wrn	156.5541	0.012103
GO:0033674~positive regulation of kinase activity	Rad50, Mre11a	143.5079	0.013196
GO:0031954~positive regulation of protein autophosphorylation	Rad50, Mre11a	95.67196	0.019732
GO:0046597~negative regulation of viral entry into host cell	Rad50, Mre11a	86.10476	0.021902
GO:0006303~double-strand break repair via nonhomologous end joining	Xrcc6, Mre11a	82.00454	0.022985
GO:0043066~negative regulation of apoptotic process	Sirt1, Mre11a, Ddx3x, Myo18a	6.085142	0.023396
GO:0071480~cellular response to gamma radiation	Xrcc6, Wrn	71.75397	0.026227
GO:0010667~negative regulation of cardiac muscle cell apoptotic process	Sirt1, Hspa8	66.23443	0.028383
GO:2001243~negative regulation of intrinsic apoptotic signaling pathway	Ddx3x, Opa1	63.78131	0.029459
GO:0071479~cellular response to ionizing radiation	Eef1d, Sirt1	55.55146	0.033753
GO:0006418~tRNA aminoacylation for protein translation	Eprs, Vars	47.83598	0.039095
GO:0007346~regulation of mitotic cell cycle	Sirt1, Rad50	46.54311	0.04016
GO:0051276~chromosome organization	Rad50, Mre11a	45.3183	0.041223
**KEGG Term**	**Genes**	**Fold Enrichment**	**p-value**
mmu03450: Non-homologous end-joining	Mre11a, Rad50, Xrcc6	148.4615	1.43E-04
mmu03440: Homologous recombination	Mre11a, Rad50	45.95238	0.039206

### RSV reduced DNA double strand breaks (DSBs) during initiation phase of reprogramming

**Figure 3:**
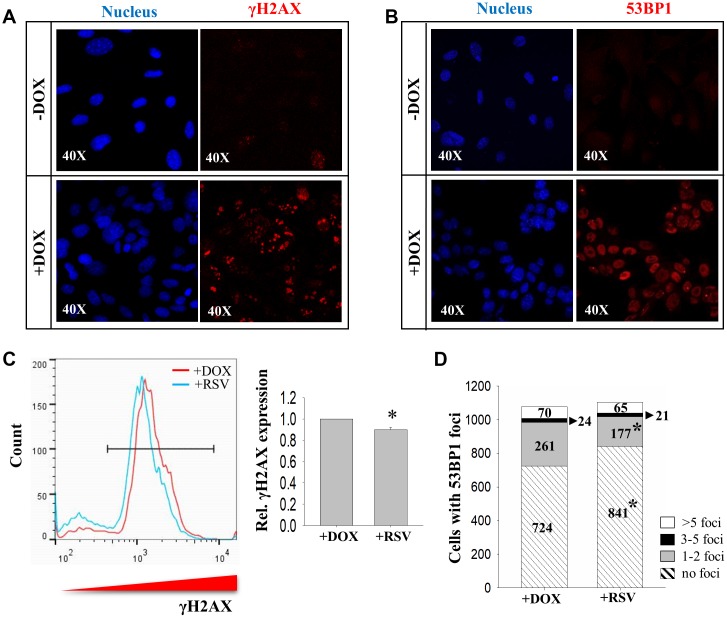
**The effects of RSV on DNA damage markers during reprogramming.** Immunocytochemistry analysis on expression of (**A**) γH2AX and (**B**) 53BP1 in 2° MEFs before (-DOX) and after (+DOX) treatment. (**C**) FACS analysis showing the effect of RSV on γH2AX expression. (**D**) The numbers of cells with more than five (>5 foci, white), three to five (3-5 foci, black), one to two (1-2 foci, grey) and no (no foci, stripped) 53bp1 foci without (+DOX) or with (+RSV) RSV treatment. (n=3; *: p<0.05 when compared to the same category; chi square test).

To verify the involvement of Sirt1 in DNA DSB repair during initiation reprogramming, DNA damage levels were assessed by measuring the levels of phosphorylated H2AX at Ser139 (γH2AX) and number of 53BP1 foci as markers of DNA DSBs. Immunostaining results demonstrated prominent increases in the number of γH2AX- (Figure 3A) and 53BP1- positive cells in the initiation phase of reprogramming (Figure 3B). Flow cytometry analysis revealed that the induced γH2AX expression was partially suppressed by treatment with *Sirt1* activator RSV (Figure 3C). Furthermore, RSV treatment also reduced the number of 53BP1 foci in DOX treated cells during initiation phase of reprogramming. The total number of cells without 53BP1 foci was significantly higher in the RSV treated group as compared to the control. Among cells with different number of 53BP1 foci, the number of cells with one to two 53BP1 foci was significantly down-regulated by the RSV treatment (Figure 3D).

### SIRT1 activator RSV rescued the inhibitory effects of WRN knockdown on reprogramming

**Figure 4:**
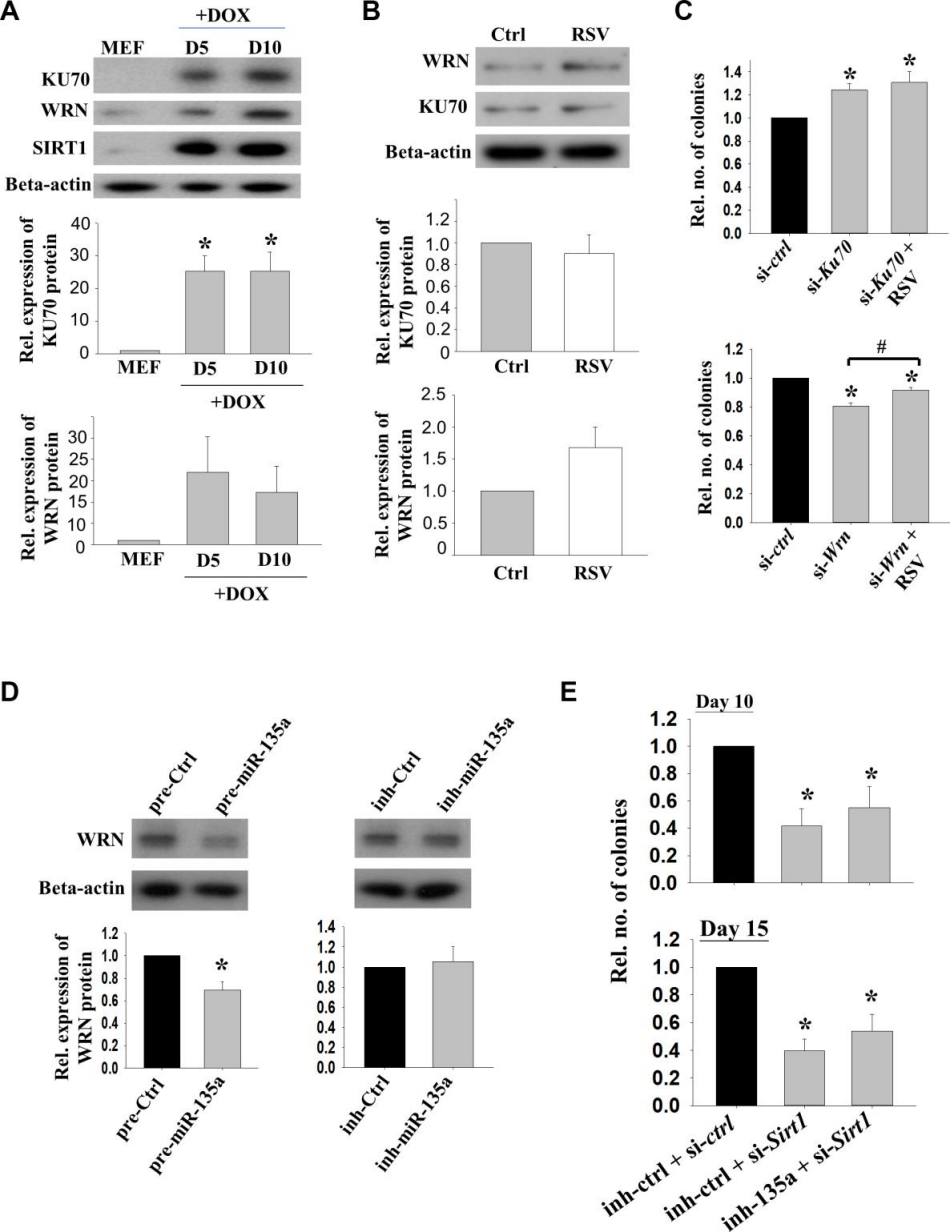
**The effects of KU70 and WRN on reprogramming efficiencies.** (**A**) The relative protein expressions of KU70, WRN and SIRT1 in reprogramming MEFs on D5 and D10 upon DOX treatment. (n=3; *: p<0.05 when compared with MEFs; one way ANOVA). (**B**) The relative protein expression levels of KU70 and WRN in 2°F MEFs without (Ctrl) or with (RSV) RSV treatment after DOX induction. (**C**) The relative number of colonies formed after transfection with *Ku70* (si-*Ku70*) or *Wrn* (si-*Wrn*) siRNA with or without the addition of RSV during the reprogramming. (n=3; *: p<0.05 as compared to siRNA control (si-ctrl); #: p<0.05 when compared with or without the addition of RSV; one way ANOVA). (**D**) The relative protein expressions of WRN in 1° MEFs after transfecting with miR-135a precursor (pre-135a) or inhibitor (inh-135a) for 72h (n=4; *:p<0.05; t-test). (**E**) The relative number of colonies formed after co-transfection with miR-135a inhibitor (inh-135a) and *Sirt1* siRNA (si-*Sirt1*) during the reprogramming. (n=6; *: p<0.05 as compared to co-transfection with miRNA inhibitor control (inh-ctrl) and siRNA control (si-ctrl); one way ANOVA).

To explore the interactions of *Sirt1* and its partners during reprogramming, protein levels of KU70 and WRN were determined in the cells on day 5 and day 10 of reprogramming. The Western blotting results confirmed that these two proteins were up-regulated along with increased SIRT1 protein levels (Figure 4A). Positive immunoreactivity of WRN was detected in the reprogramming cells, but not in MEFs without DOX treatment [Supplementary Figure 3]. However, the expressions of KU70 and WRN were not affected by RSV treatment during the initiation phase of reprogramming (Figure 4B).

Next, we studied the roles of *Sirt1* interacting proteins on reprogramming. 2° MEFs were transfected with *Ku70* or *Wrn* siRNA. The number of colonies formed was significantly decreased after the transfection of *Wrn* siRNA. Surprisingly, transfection of *Ku70* siRNA significantly enhanced the reprogramming efficiency. The addition of RSV partly rescued the inhibitory effect of *Wrn* siRNA on reprograming efficiency (Figure 4C). Western blotting result showed that WRN protein expression was significantly reduced upon the treatment of miR-135a precursor, while no effect was observed when miR-135a inhibitor was used (Figure 4D). The effects of *Sirt1* knockdown in the presence of miR-135a inhibitor was followed. The data showed that *Sirt1* knockdown significantly inhibited the reprogramming efficiency (p<0.05). Though not statistically significant, the addition of miR-135a inhibitor partially rescued the effect of *Sirt1* knockdown (Figure 4E).

## DISCUSSION

Reprogramming of somatic cells into pluripotent cells is a long process involving global genetic and epigenetic remodeling [18]. miRNAs have been reported to modulate reprogramming efficiency [19]. Epigenetic regulators and transcription factors are major miRNA targets [20]. We have reported the modulation effect of miR-34a via histone deacetylase *Sirt1* on cellular reprogramming [8]. In an attempt to further delineate the involvement of miRNA on epigenetic regulators during cell fate determination, we searched for other miRNAs that might also regulate *Sirt1*. In this study, we employed both 1° and 2° MEFs reprogramming system to confirm the effects of miR-135a and *Sirt1* on cellular reprogramming. As the reprogramming efficiency of 1° MEFs suffers from batch-to-batch variation upon the introduction of lentiviral vector, the DOX-inducible 2° MEFs having the advantage of robust reprogramming efficiency was included in this study.

Here, we demonstrated for the first time that miR-135a affected the reprogramming efficiency of somatic cells into pluripotent cells. *Sirt1* has been reported to be regulated by miR-34a [8] and miR-195 during cellular reprogramming [13]. In this study, we found that *Sirt1* is the main target of miR-135a as more than half of the SIRT1 protein level was reduced upon the overexpression of miR-135a precursor. In fact, *Sirt1* was a target of miR-135a in mESCs [21] and cancer cells [15]. Inhibition of miR-135a enhanced proliferation of MEFs, which might be related to the role of Sirt1 in opposing DNA damage.

To decipher the mechanistic roles of *Sirt1*, we identified SIRT1 interacting proteins in the initiation phase of reprogramming. Interestingly, the GO terms and KEGG pathways enriched for the 21 SIRT1 interacting proteins were highly related to DNA DSB repair and DNA damage repair. Our present data demonstrated increased levels of DNA damage markers, γH2AX and 53BP1 foci [22, 23], indicating the induction of DNA damage upon reprogramming. The results are in line with other studies reporting the induction of γH2AX expression and DNA DSB after ectopic expression of reprogramming factors [24]. Another study also used γH2AX and 53BP1 foci staining as the markers to demonstrate the increase of DSB during initiation phase of reprogramming [25]. Nonhomologous end joining (NHEJ) activity is higher in human iPSCs when compared to their parental cells, suggesting the level of DSB declines upon stabilization of iPSC [26]. We found in this study that *Sirt1* activator RSV significantly lowered the expression of γH2AX and reduced the number of 53BP1 foci during reprogramming, suggesting that *Sirt1* increased reprogramming efficiency partly through DNA damage repair. The data supports the presence of miR-135a-*Sirt1*-DSB repair proteins during the initiation phase of reprogramming. On the other hand, we should note that DNA structures can be modified by a number of methylation and alkylation agents, like S-adenosylmethionine (SAM) and azinomycins, leading to gene mutation [27–29]. The role of resveratrol used in this study on DNA strand modification required further investigation.

Excessive accumulation of DSBs led to cellular growth arrest, apoptosis and mutations. Among the SIRT1 interacting proteins, four DSB repair proteins (KU70, WRN, RAD50 and MRE11A) were identified. While *Ku70* and *Wrn* are related to NHEJ, *Rad50* and *Mre11a* are related to homologous recombination (HR). NHEJ and HR are two major DNA DSB repair pathways that play critical roles in iPSCs generation. While inhibition of HR pathway impaired [24], overexpression of the HR gene, *Rad51*, significantly enhanced reprogramming efficiency [30]. On the other hand, the mutation of *Lig4*, one of the enzymes responsible for NHEJ, led to inhibition of reprogramming efficiency [31]. DNA damage repair is also involved in epigenetic regulation. It was reported that histone deacetylase inhibitor enhanced the expression of *Ku70* and *Mre11a* in mouse embryos after somatic cell nuclear transfer [32]. However, the exact roles of the above 4 SIRT1 interacting proteins during reprogramming process are unclear. As NHEJ is the major and primary pathway for the DSB repairs in eukaryotes [33], *Ku70* and *Wrn* were first selected for the current study.

*Ku70* is the main component of the NHEJ pathway [34]. Unexpectedly, *Ku70* knockdown led to enhanced reprogramming efficiency in the current study. *Ku70* was reported to have NHEJ independent functions. The knockout of *Ku70* stabilizes β-catenin [35] which induces iPSC formation by interacting with reprogramming factors (*Klf4*, *Oct4*, and *Sox2*) during the initial stage of reprogramming [36]. In addition, *Ku70* was reported to repress *p53* expression [37], which we [8] and others [38] demonstrated to serve as a barrier to reprogramming. In this connection, the observed induction of iPSC formation upon *Ku70* knockdown might be a combination of NHEJ and non-NHEJ effects, which warrant further detail investigation.

In this study, we also investigated the effects of *Wrn* on iPSC formation during initial phase of reprogramming. *Wrn* belongs to RecQ DNA helicase family and participates in DNA DSB repair by NHEJ [39]. As expected, it was upregulated upon initiation of reprogramming and its knockdown inhibited reprogramming efficiency. Indeed, *Wrn* silencing was shown to induce DNA damage through *p53*, accelerate cellular senescence and inhibite the proliferation of human fibroblasts [40]. On the other hand, SIRT1 has been previously reported to deacetylate WRN and promote its translocation for DNA repair [41, 42]. Another study also reported SIRT1 stabilized WRN protein through deacetylation, which in turn prevented WRN from proteasome degradation. Inhibiting SIRT1 deacetylase activity led to downregulation of WRN protein levels [43]. Though RSV had no effect on modulating WRN protein level, it partially rescued the inhibitory effect of *Wrn* knockdown on iPSC formation, linking the *Sirt1* induced iPSC formation to its interaction with WRN via DNA damage repair. WRN also interacts physically with KU70/80 heterodimer [44]. The fact that SIRT1 interacted with both KU70 and WRN suggested the involvement of SIRT1 in a complex of DSB repair proteins during the initiation phase of reprogramming, leading to increase in DNA repair and improvement of reprogramming efficiency. Furthermore, WRN protein expression was significantly reduced upon the treatment of miR-34a precursor. However, RSV had no effect on the expressions of miR-135a, suggesting that miR-135a is an upstream regulator of *Sirt1* and *Wrn* during reprogramming. Meanwhile, the addition of miR-135a inhibitor partially rescued the effect of *Sirt1* knockdown on iPSC formation also suggested the involvement of miR-135a-*Sirt1* during reprogramming. Although the result was not statistically significant, it might be due to the efficient knockdown of *Sirt1* that no prominent effect could be observed in the presence of miR-34a inhibitor. Further approach by force expression of *Sirt1* in the presence of miR-34a precursor warrants investigation.

Apart from KU70 and WRN, MRE11-RAD50-NBS1 (MRN) complex is also important in the HR pathway mediated DSB repair [45]. The fact that MRE11 and RAD50 were also within the complex with SIRT1 during the initiation phase of reprogramming suggested the interaction of MRN complex with SIRT1 during cellular reprogramming. However, the functional roles of molecules in MRN complex require further studies.

Histone deacetylation is generally linked to condensed chromatin structure and silenced transcription [46]. Chromatin remodelling was reported during reprogramming. For examples, H3K4me3 induced by TRX protein and histone acetylation for active transcription of pluripotent genes induced by P300 protein were reported [47]. The fact that *Sirt1* belongs to histone deacetylase prompt to the postulation that *Sirt1* might induce macroscopic chromatin rearrangement during reprogramming. The specific roles of *Sirt1* in regulating chromatin structures during reprogramming requires further studies.

In comparison with our previous data [8], both miR-34a and miR-135a inhibitors stimulated a 2-fold increase in iPSC colony formation after 10 days of reprogramming. After 15 days of reprogramming, miR-34a inhibitor stimulated a 8-fold while miR-135a inhibitor stimulated a 2-fold increase in iPSC colony formation as compared to the controls. The data suggests that miR-34a is more efficient in affecting the reprogramming. It is possible that different pathways are involved in the regulation of reprogramming by miR-34a and miR-135a; while miR-34a regulated *Sirt1* enhances iPSC formation through deacetylation of p53, leading to upregulation of *Nanog* while downregulation of *p21* levels [8], miR-135a regulated *Sirt1* enhances iPSC formation through DNA damage repair. It is worthwhile to study the combined effects of miR-135a and miR-34a.

To conclude, our results showed that miR-135a inhibited reprogramming efficiency partly through suppressing *Sirt1*. We also uncovered the unprecedented roles of SIRT1-interacting proteins, KU70 and WRN, in reprogramming.

## MATERIALS AND METHODS

### Mouse ESC culture and differentiation

Mouse ESC (mESC), L4 was obtained from the Transgenic Core Facility, Department of Biochemistry, The University of Hong Kong. L4 and mouse iPSCs were cultured as described [8]. Briefly, the cells were cultured in mESC medium and passaged every 2 to 3 days. They were differentiated into EBs using hanging drop method.

### Reprogramming of MEF to iPSCs

MEF were derived from 14.5dpc ICR (CD-1^®^) mouse embryos according to a published protocol [48]. MEFs were seeded onto 0.1% gelatin (Thermo Fisher Scientific, New York, USA) coated plates at a density of 1.33x10^4^ cells/cm^2^. For reprogramming, primary MEFs (1° MEFs) were transduced with lentiviral vectors TetO-FUW-OSKM (Addgene #20321) and FUW-M2rtTA (Addgene #20342) at different multiplicity of infection (MOI) for 24 hr. The MEFs were re-plated 72 hr post-infection at a density of 1666 cells/cm^2^. Reprogramming was induced by addition of doxycycline (DOX; 1.5μg/ml; Sigma-Aldrich, Missouri, USA) to mESC medium. The number of iPSC colonies were assessed on the days according to the design of each experiment. All animal experiments were performed in accordance with the guidelines on animal care and with prior approval by the Committee on the Use of Live Animals in Teaching and Research (CULATR), The University of Hong Kong.

Secondary PB-iPSC-derived mouse embryonic fibroblasts (2° MEFs) containing the DOX-inducible reprogramming factors were obtained from Prof. A. Nagy (Samuel Lunenfeld Research Institute, Mount Sinai Hospital, Toronto, Canada) and isolated as previously described [2]. 2° MEFs were seeded at a density of 833 cells/cm^2^ and were induced to reprogram in mESC medium supplemented with 1.5 μg/ml of DOX.

### Small RNA transfection and RSV treatment

1° and 2° MEFs were transfected prior to reprogramming as previously described [8]. Briefly, the cells were transfected with si-*Sirt1*, si-*Ku70* and si-*Wrn* at a concentration of 100 nM (Santa Cruz Biotechnology, Texas, USA). Scrambled siRNA was used as control. Precursor of miR-135a and random sequence precursors (control) at a concentration of 50 nM (Thermo Fisher Scientific). miRCURY LNA inhibitor of miR-135a and random sequence inhibitors (control) at a concentration of 50 nM (Exiqon, Vedbaek, Denmark) were also transfected into the cells. RSV (Sigma-Aldrich) was treated at a concentration of 1 μM.

### Quantitative polymerase chain reaction (qPCR) and western blotting analysis

Total RNAs were extracted by the mirVana PARIS Kit (Thermo Fisher Scientific) and converted to cDNA by the TaqMan Reverse Transcription kit (Thermo Fisher Scientific). Real time quantitative PCR (qPCR) was performed in an Applied Biosystems 7500 Real-Time PCR System (Thermo Fisher Scientific) using the TaqMan Gene Expression Assay. Quantifications were determined by the 2^-ΔΔCT^ method. The mRNA levels were normalized with the endogenous 18S ribosomal RNA.

For protein analysis, cells were lysed in cell lysis buffer (Thermo Fisher Scientific) containing protease inhibitors (Sigma-Aldrich). Equal amounts of denatured proteins were separated by electrophoresis in 10% sodium dodecyl sulfate-polyacrylamide gel electrophoresis (SDS-PAGE), and transferred to polyvinylidene difluoride membrane (Millipore, Massachusetts, USA). The membranes were incubated successively with primary antibodies against SIRT1 (Cell Signaling), WRN (Abcam, Cambridge, United Kingdom), KU70 (Abcam) and β-ACTIN (Sigma-Aldrich). Appropriate horseradish peroxidase (HRP) conjugated secondary antibodies (GE Healthcare, Illinois, USA) was followed, and the membranes were developed in X-ray films using WesternBright ECL Kit (Advansta, California, USA). The quantification of protein bands was analyzed by ImageJ software.

### Immunocytochemistry and alkaline phosphatase staining

Cells were fixed in 4% paraformaldehyde (Sigma-Aldrich). After permeabilization with 0.1% Triton X-100 (Sigma-Aldrich), they were incubated with appropriate blocking solution (10% normal goat serum) followed by primary antibodies against SSEA-1 (Thermo Fisher Scientific), OCT4 (Santa Cruz Biotechnology), NANOG (Millipore), γH2AX (BD Biosciences, California, USA), WRN (Abcam) and 53BP1 (Santa Cruz Biotechnology) at 4°C overnight. The cells were then incubated with fluorescent-conjugated secondary antibodies (Thermo Fisher Scientific). The nucleus was stained with Hoechst 33258 (Thermo Fisher Scientific). Images of the staining cells were captured using a confocal microscope (LSM 700, Carl Zeiss AG, Oberkochen, Germany) at the Faculty Core Facility, The University of Hong Kong.

### Fluorescence activated cell sorting (FACS) and flow cytometry analysis

Population of GFP positive cells containing DOX-inducible reprogramming factors were sorted out from 2° MEFs using a BD FACSAria I Cell Sorter (BD Biosciences). For quantification of DNA damage level, the expression of γH2AX in reprogramming cells was analyzed by flow cytometry. Briefly, 2° MEFs after reprogramming for 5 days were dissociated into single cells. After fixation and permeabilization, the cells were incubated with anti-γH2AX antibody for 30 min at room temperature, followed by goat anti-rabbit-Alexa Fluor 568 for 30 min. The proportion of PE positive cells was analyzed by a BD LSR Fortessa Analyzer (BD Biosciences) and the FlowJo software at the Faculty Core Facility, The University of Hong Kong.

### Immunoprecipitation

Cell extract for immunoprecipitation was prepared from the population of GFP positive 2° MEFs cultured in the presence of DOX for 5 days. The cells were lysed by the CytoBuster™ Protein Extraction Reagent (Novagen, Millipore) in the presence of protease inhibitor. The lysate (>500 μg) was incubated with anti-SIRT1 antibodies (Millipore) or normal rabbit IgG (Millipore) as control at 4°C overnight. The mixture was then incubated with protein-G-sepharose beads (Millipore) at 4°C for 90 min. Following centrifugation, the beads were washed with PBS and resuspended in 20 μl of PBS ready for SDS-PAGE analysis, western blotting and silver staining.

### Silver staining and mass spectrometry analysis

Protein samples after SDS-PAGE were stained using the PlusOne™ silver staining kit (GE Healthcare). The stained protein bands of interest were identified by 1D-LC-ESI-Ion Trap-mass spectrometry analysis in the Proteomic Laboratory for System Biology Research in Hong Kong Baptist University.

### Proliferation assay

Proliferation of MEFs was determined by the CyQUANT® NF Cell Proliferation Assay Kit (Thermo Fisher Scientific). The fluorescence readings were measured at an excitation of 485 nm and an emission of 530 nm using a Tecan Infinite F200 plate reader (Tecan Life Sciences, Männedorf, Switzerland).

### Bioinformatics analysis

Gene ontology analysis was performed using the Database for Annotation, Visualization and Integrated Discovery (DAVID, version 6.8).

### Statistical analysis

Data were analyzed and plotted using the SigmaPlot software (Aspire Software International). Statistical analysis was performed by t-test, Rank Sum test or One Way ANOVA as appropriate. A p-value < 0.05 was considered as statistical significance.

## Supplementary Material

Supplementary Figures
