# Supplementary material for: Sirt1 is regulated by miR-135a and involved in DNA damage repair during mouse cellular reprogramming
Source: Aging (Albany NY). 2020 Apr 26;12(8):7431–47. doi: 10.18632/aging.103090 (PMC7202538; doi:10.18632/aging.103090)
Supplement: Supplementary Figures [file aging-12-103090-s001..pdf]

SUPPLEMENTARY FIGURES

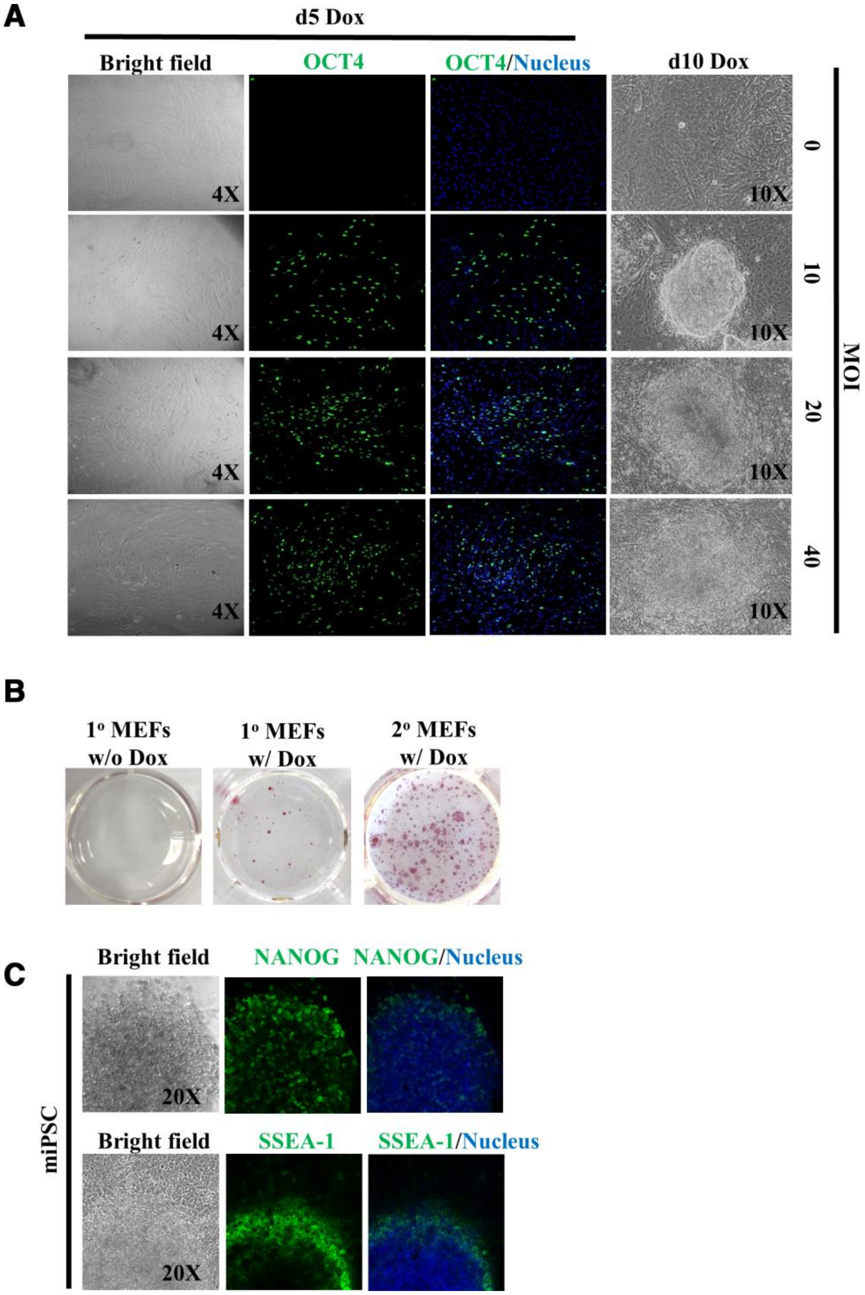

Supplementary Figure 1. Establishment of 1° and 2° reprogramming systems from MEFs. (**A**) Immunocytochemistry analysis of OCT4 (green) in 1° MEFs transduced with lentivirus at different multiplicity of induction (MOI). The nuclei were stained with DAPI (blue). (**B**) Alkaline phosphate staining on iPSC colonies formed after 15 days of DOX treatment in both 1° and 2° MEFs. (**C**) Immunocytochemistry analysis of NANOG and SSEA-1 in iPSC colonies formed from 1° MEFs after 15 days of DOX treatment.

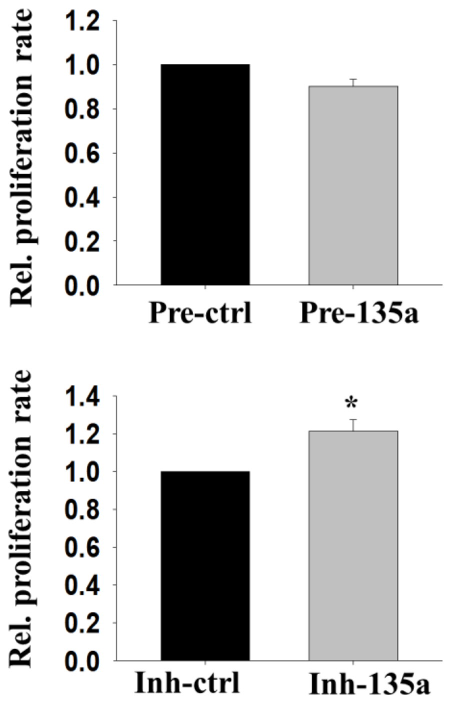

Supplementary Figure 2. miR-135a affected proliferation rates of MEFs. The relative proliferation rates of 1° MEFs after treatment with pre-135a or inh-135a for 24h (n=4; *:p<0.05; t-test).

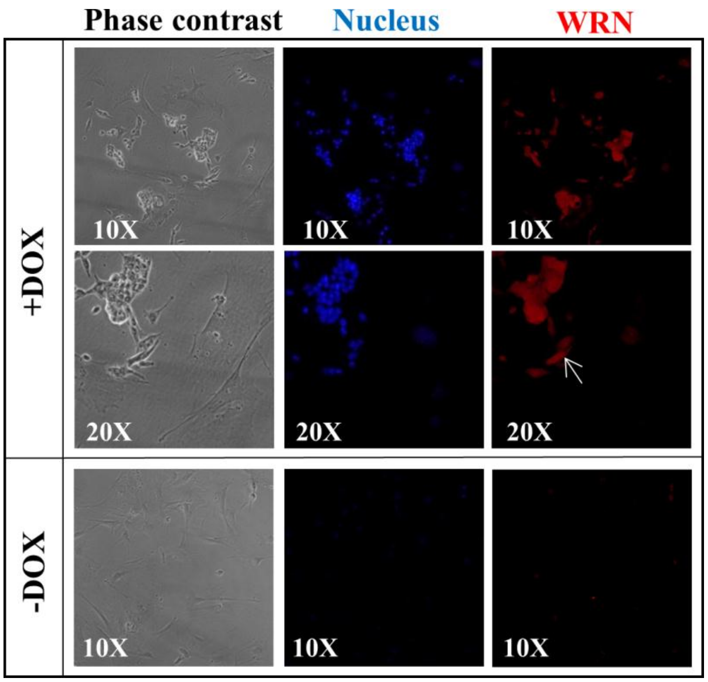

Supplementary Figure 3. Expression of Wrn during reprogramming. Immunocytochemistry analysis on WRN protein in 2° MEFs with (+DOX) or without (-DOX) DOX induction for 5 days. White arrow: reprogramming MEFs.
